# Clinical Trial of an Oral Live Shigella sonnei Vaccine Candidate, WRSS1, in Thai Adults

**DOI:** 10.1128/CVI.00665-15

**Published:** 2016-07-05

**Authors:** Punnee Pitisuttithum, Dilara Islam, Supat Chamnanchanunt, Nattaya Ruamsap, Patchariya Khantapura, Jaranit Kaewkungwal, Chatporn Kittitrakul, Viravarn Luvira, Jittima Dhitavat, Malabi M. Venkatesan, Carl J. Mason, Ladaporn Bodhidatta

**Affiliations:** a Department of Clinical Tropical Medicine and Vaccine Trial Centre, Faculty of Tropical Medicine, Mahidol University, Bangkok, Thailand; b Armed Forces Research Institute of Medical Sciences, Bangkok, Thailand; c Faculty of Tropical Medicine, Mahidol University, Bangkok, Thailand; d Department of Enteric Infections, Bacterial Diseases Branch, Walter Reed Army Institute of Research, Silver Spring, Maryland, USA; University of Maryland School of Medicine

## Abstract

Live attenuated Shigella sonnei vaccine candidate WRSS1, previously tested in U.S. and Israeli volunteers, was evaluated in a population of adult Thai volunteers in which the organism is endemic. In a randomized placebo-controlled, double-blind design, inpatient participants received a single oral dose of 1.6 × 10^4^ CFU of WRSS1. The vaccine was generally well tolerated, with equal numbers of vaccinees and placebo controls showing mild symptoms. Only 3 of 13 vaccinees (23%) had culture-positive stools, while a total of 9 vaccinees were positive by PCR. Lack of vaccine shedding in volunteers correlated with lack of clinical symptoms and immune responses, just as the duration of fecal shedding correlated directly with stronger immune responses. Two months following immunization, 10 vaccinees and 10 newly recruited naive controls received a challenge dose of 1,670 CFU of virulent S. sonnei strain 53G. This dose had previously demonstrated a 75% attack rate for dysentery in Thai volunteers. However, in this study the attack rate for dysentery in naive controls after challenge was 20%. Based on clinical record summaries, 3 vaccinees and 5 naive controls experienced clinically relevant illness (diarrhea/dysentery/fever/shigellosis), and a 40% vaccine efficacy was calculated. When these data are compared to those for the performance of this vaccine candidate in more naive populations, it is clear that a single oral dose of WRSS1 at 10^4^ CFU failed to achieve its full potential in a population in which the organism is endemic. Higher doses and/or repeated immunizations may contribute to improved vaccine shedding and consequent elevation of protective immune responses in a population in which the organism is endemic. (The study has been registered at ClinicalTrials.gov under registration no. NCT01080716.)

## INTRODUCTION

Shigellosis or bacillary dysentery is an important cause of morbidity and mortality among vulnerable groups, including travelers and children in less developed countries (1–4). In a recent global enteric multicenter study (GEMS), Shigella, rotavirus, Cryptosporidium, and enterotoxigenic Escherichia coli expressing heat-stable toxin were shown to be the 4 main causes of moderate to severe diarrhea in children less than 5 years of age (2). The GEMS results indicated that from 12 to 59 months of age, Shigella was an important bacterial pathogenic cause of moderate to severe diarrhea. Shigella is a risk factor for mortality, poor physical growth, and impaired cognitive development. The distribution of Shigella species and serotypes is region specific and changes over time in resource-poor regions of the world (5). In Thailand, in the past, S. flexneri (79%) and S. sonnei (15%) have been the most commonly isolated Shigella species (1, 3). However, more recent estimates have reversed this trend and currently S. sonnei predominates (84%), with the rest being serotypes of S. flexneri (5, 6). This shifting trend in distribution of Shigella serotypes is similar to what is seen in Vietnam and Malaysia (7, 8).

The burden of shigellosis is greatest in poor countries. Shigella is easily transmitted in crowded and unhygienic environments (9, 10); contaminated water supplies and poor sanitation are the primary risk factors for shigellosis worldwide (5, 11–13). Additionally, there is a correlation between socioeconomic status and the risk for shigellosis (5). The increased risk of exposure to Shigella was reflected in a high prevalence of antibodies to Shigella in Israel, where Shigella antibodies in young adults were associated with a low socioeconomic background (6, 14). In Bangkok, Thailand, the adult population may have had prior exposure to S. sonnei, as demonstrated by S. sonnei-specific serum IgG antibodies. Natural exposure to Shigella generally produces short-lived serotype-specific protection.

Control of shigellosis by hygienic measures is often difficult because of the very low infectious dose, and prevention by active vaccination may be optimal, presenting an important measure to reduce rates of morbidity and mortality due to Shigella infections. Currently there are no commercially licensed Shigella vaccines outside China, although several decades of research have resulted in the evaluation of a variety of live, killed, and subunit vaccine candidates. In China, a live, oral, noninvasive, bivalent vaccine expressing O antigens of S. sonnei and S. flexneri 2a has been used in adults as a licensed product (15).

WRSS1, a live attenuated S. sonnei vaccine candidate, is principally attenuated by the loss of the virulence plasmid-borne *virG* (or *icsA*) function, eliminating the ability of the pathogen to spread intercellularly. In animal and human studies, *virG* (*icsA*) mutants are significantly attenuated. WRSS1 was constructed using the stable S. sonnei strain Moseley as the parental strain (16). It was manufactured in the mid-1990s as a lyophilized product at the Walter Reed Army Institute of Research (WRAIR) Pilot Bioproduction Facility (PBF) in Silver Spring, MD, USA. WRSS1 is invasive in HeLa cells but negative in the plaque assay and the Sereny test. WRSS1 was both immunogenic and protective in the guinea pig model (16). Recent genomic sequencing analysis has indicated that an additional chromosomal deletion of 82 kb occurred spontaneously during the construction of WRSS1 (J. R. Ticehurst and M. M. Venkatesan, unpublished). This chromosomal deletion does not affect cellular invasion or intracellular replication. WRSS1 is invasive in HeLa cells and can multiply intracellularly, and complementation of WRSS1 with a *virG* (*icsA*) gene restores its ability to be plaque positive and Sereny positive in guinea pigs (16, 17).

WRSS1 completed phase I trials in U.S. and Israeli volunteers and was shown to be safe, with high rates of fecal shedding and robust immunogenicity (18, 19). No evidence of transmission was observed in participants cohabitating with vaccinees during the trial in Israel. So far, no efficacy studies have been conducted with WRSS1. The objective of this study was to assess the safety, immunogenicity, and efficacy of a single dose of WRSS1 in Thailand, where S. sonnei is endemic.

## MATERIALS AND METHODS

This study was designed as an inpatient trial and was approved by the Human Subjects Research Review Board of the U.S. Army Medical Research and Materiel Command (USAMRMC), the Ethical Review Committee for Research in Human Subjects, Ministry of Public Health, Thailand, and the Ethics Committee of the Faculty of Tropical Medicine, Mahidol University.

### Study design.

Healthy male or female Thai adults, between the ages of 20 and 40, were recruited from the Bangkok Metropolitan area and its periphery. Written informed consent was obtained from all volunteers. Inclusion criteria included normal bowel habits (defined as fewer than 3 stools per day and greater than 1 per 2 days), being negative for serum and urine β-human chorionic gonadotrophin (female volunteers), and having baseline anti-S. sonnei LPS IgG antibody titers of ≤1:800 (data not shown). Potential volunteers were excluded if they had significant health problems, including arthritis or being positive for hepatitis B surface antigen; had prolonged prothrombin time (PT), mild anemia, leukocytosis, external hemorrhoids, hypertension, elevated bilirubin, high urine protein/glucose, elevated aspartate transaminase (AST) and/or alanine transaminase (ALT), or eosinophilia; or were human leukocyte antigen (HLA) B27 positive (HLA B27-positive individuals are considered to be at a higher risk for reactive arthritis after a Gram-negative infection).

**FIG 1:**
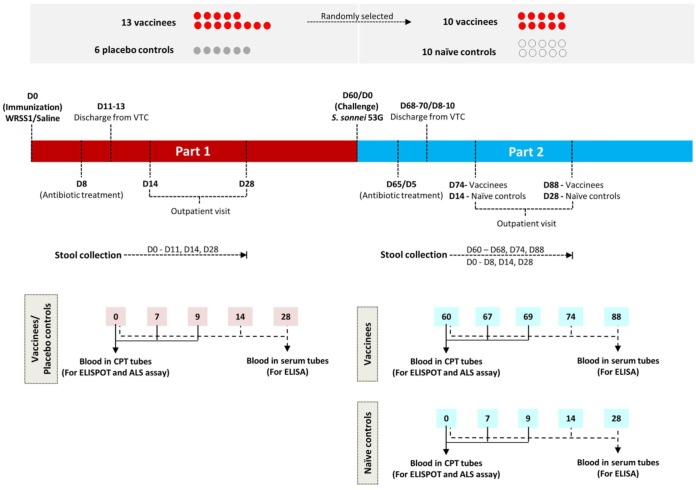
Study outline for oral live S. sonnei vaccine candidate WRSS1 immunization and wild-type S. sonnei 53G challenge of Thai adults.

The study outline is shown in Fig. 1. The first phase of the study was carried out as a randomized, double-blind, placebo-controlled trial to evaluate the safety and immunogenicity of WRSS1. The 10^4^-CFU dose was based on the safety dose determined from studies of U.S. and Israeli volunteers (18, 19). Fourteen vaccinees and 6 controls (placebo) were enrolled; however, one subject was excluded on study day 0 because of an episode of diarrhea. Volunteers were admitted to the quarantine ward at the Mahidol University Vaccine Trial Centre (VTC), Bangkok, Thailand. After a 90-min fasting period, 13 vaccinees drank 150 ml of a bicarbonate solution (2 g in 150 ml of sterile water) to neutralize gastric acidity prior to receiving a single oral dose of 1.6 × 10^4^ CFU of WRSS1 that was suspended in 30 ml of sterile water. Six placebo controls received 1 ml of sterile saline in 30 ml of sterile water. The second phase of the study was an efficacy assessment involving 10 randomly selected immunized volunteers from the first phase of the study who were challenged 60 days after vaccination with a 1,670-CFU dose of wild-type S. sonnei strain 53G. In a previous study, this challenge dose had demonstrated a 75% attack rate for dysentery in Thai adults (20). Additionally, 10 newly recruited naive volunteers also were recruited as controls and challenged with the 53G strain. Blood and stool samples were collected during both phases of the study, and the volunteers were assessed daily for reactogenicity and adverse events (AE). Based on clinical signs and symptoms associated with the vaccine, any AE were graded as mild (symptoms were present but the subject was able to perform daily activities), moderate (the subject's activities were reduced, but some could still be performed), and severe (the subject's activities were markedly reduced).

The volunteers were treated with ciprofloxacin on study day 8 after WRSS1 vaccination and on study day 5 after 53G challenge. In the first phase of the study, volunteers were discharged from the VTC on study days 11 to 13 after antibiotic treatment, while volunteers in the efficacy phase of the study were discharged on study days 8 to 10 after 53G challenge when volunteers were symptom free, completed antibiotic treatment, and had no shedding in stool. They returned for outpatient visits on days 14 and 28 after vaccination and after challenge and were contacted by telephone for a final assessment on study day 60 following vaccination as well as challenge.

### Stool grading and clinical endpoints of disease.

All stools were examined for gross and occult blood and then graded as to consistency with the following scores: 1 to 2, hard to soft (not affecting normal activities and causing only slight discomfort); 3, thick liquid (diarrhea); 4 to 5, opaque watery liquid to clear watery liquid (diarrhea). Diarrhea was defined as 2 or more grade 3 to 5 stools (total volume of ≥200 ml) or a single grade 3 stool or higher with stool volume of ≥300 ml within 48 h. Dysentery was characterized by grade 3 stools or higher with gross blood (confirmed by study personnel or ward nurse). Shigellosis was defined as diarrhea and/or dysentery accompanied by fever (temperature of >38.3°C or 100.94°F), one or more severe intestinal symptoms (abdominal pain, nausea, vomiting, and anorexia), and shedding of S. sonnei. Clinical endpoints of disease were defined as diarrhea, dysentery, and/or fever.

### Preparation of WRSS1.

WRSS1 (lot no. 0451) was manufactured in 1997, is stored as lyophilized vials at −80°C, and has maintained both its viability and the stability of its form I phenotype. The form I phenotype refers to smooth-edged, small, round colonies and is indicative of the presence of the large virulence plasmid (VP) that contains the O-antigen genes in S. sonnei. Loss of the VP results in larger, flat, and irregular-edged form II colonies that are noninvasive and avirulent. For the trial, frozen vials of WRSS1 on dry ice were sent from WRAIR PBF to the Armed Forces Research Institute of Medical Sciences (AFRIMS), Bangkok, Thailand. On the immunization day, a frozen vial of WRSS1 was removed from the −80°C freezer and transported on wet ice to prepare the dose at the VTC. The lyophilized vial was rehydrated with 5 ml of sterile water for injection (SWI). While it was sitting on wet ice, the WRSS1 suspension was gently swirled until it was completely dissolved. The suspension was further diluted with sterile normal saline to obtain a WRSS1 dose of 10^4^ CFU/ml. Within 15 min before immunization, 1 ml of the WRSS1 suspension was dispensed into a cup. After visual confirmation of the presence of vaccine in each individual cup, 30 ml of chilled SWI was added into 1 ml of the WRSS1 dose (10^4^ CFU/ml) and then thoroughly mixed. This 31-ml WRSS1 suspension was orally administered to each volunteer. The actual inoculum dose was evaluated by viable-cell counting of the reconstituted vaccine on a Trypticase soy agar (TSA) plate. The placebo controls in the first phase of the study received 1 ml of saline combined with 30 ml of SWI.

### Preparation of 53G challenge dose.

A production cell bank (BPR-328-00; lot no. 0599) of S. sonnei 53G that was manufactured under cGMP by the WRAIR PBF in 1998 was transferred to AFRIMS and was the starting material for the production of the AFRIMS stock. For this study, the S. sonnei 53G AFRIMS stock was plated on a Congo red agar plate that was incubated at 37°C for 14 to 18 h. Congo red-positive colonies with the form I phenotype were subjected to slide agglutination with S. sonnei form I antiserum (Denka Seiken, Tokyo, Japan). Agglutination-positive colonies were suspended in phosphate-buffered saline (PBS) prior to their being spread evenly on TSA plates. After incubation overnight at 37°C, the bacterial lawn was harvested in PBS and adjusted to an optical density at 600 nm (OD_600_) of 1.0 to 1.5. This bacterial suspension was further diluted with PBS to obtain an OD_600_ of 0.16, which corresponds to approximately 1.6 × 10^8^ CFU/ml. The S. sonnei 53G inoculum dose of 1,600 CFU/ml was prepared by serially diluting the bacterial suspension to 1.6 × 10^3^ CFU/ml using normal saline. One milliliter with 1,600 CFU was suspended in 30 ml of SWI for oral administration to each volunteer. The actual challenge inoculum dose was determined by performing viable-cell counts on TSA plates. The virulence of the challenge strain was evaluated by the plaque assay.

### Stool culture for detection of WRSS1 and 53G.

Stool samples from both phases of the study were plated on Hektoen enteric agar and incubated overnight at 37°C. Up to 10 non-lactose-fermenting (NLF) colonies were selected and tested by agglutination with S. sonnei antiserum (Denka Seiken, Tokyo, Japan) as previously described. If none of the NLF colonies showed agglutination, a representative colony was tested by standard biochemical reactions (20).

### Detection of Shigella in stool samples.

DNA templates were prepared from fecal samples using a QiaAmp stool purification kit (Qiagen Inc., Valencia, CA, USA). Templates were used in real-time PCR (RT-PCR) using an ABI 7900 sequence detection system instrument (Applied Biosystems, Foster City, CA). For Shigella detection an *ipaH* gene-based RT-PCR assay was utilized (21). The primers and probes used were the following: *ipaH*-U1 forward (F), CCTTTTCCGCGTTCCTTGA; *ipaH*-L1 reverse (R), CGGAATCCGGAGGTATTGC; and *ipaH*-P1 probe, VIC-CGCCTTTCCGATACCGTCTCTGCA-6-carboxytetramethylrhodamine (TAMRA). For PCR detection of WRSS1 in stool samples, the primers used were the following: F, 5′CGT AGG TAG GTA GCA TGC GT3′; R, 5′CAG AAC CTT CAT GGA GTA TTA ATG A3′; and probe, 6-carboxyfluorescein (FAM)-TAA TGC TAT CCA CAT CAC TGG TGG AAA CAA-TAMRA. For PCR detection of S. sonnei 53G in stools, aliquots of approximately 0.5 ml of frozen stool or one rectal swab were saved in a transporting buffer to prepare the DNA template. For the 53G wild-type strain a *virG* gene-based conventional PCR assay was utilized. The primers used were the following: forward, BA114a, 5′GCG CGT CGA CCC AAT GTT CAC AGG GG3′; reverse, BA117, 5′GGC GCG AGC TCA ACT GTC CCC ATT TTT AG3′. PCR was followed by gel electrophoresis to detect the presence of a 1,644-bp amplified product (16, 20).

### Fecal extraction for ELISA.

Stool samples were thawed, and 1 g of stool was added with 2 ml of cold PBS (containing 1 mg/ml EDTA, 0.2 mg/ml trypsin soybean inhibitor, 1 mg/ml bovine serum albumin, 17.5 mg/ml phenylmethylsulfonyl fluoride, and 0.05% Tween 20) as previously described (22). Samples were incubated for 20 to 25 min on wet ice with intermittent vortexing every 5 min. After centrifugation at 20,000 × *g* for 30 min at 4°C, the clear supernatant was filtered and stored at −70°C until it was used for fecal IgA antibody assay by enzyme-linked immunosorbent assay (ELISA).

### Serum and fecal antibodies against S. sonnei LPS and Invaplex.

Serum and stool immune responses were measured against S. sonnei lipopolysaccharide (LPS) (AI BioTech, LLC., Richmond, VA, USA) and S. sonnei Invaplex-50 protein antigens (INV; INV contains mostly LPS and the invasion plasmid antigens; received from E. Oaks, Department of Enteric Infections, WRAIR, Silver Spring, MD, USA). During the vaccination phase, blood was collected on days 0, 14, and 28 and tested for IgA, IgM, and IgG antibodies to S. sonnei LPS and S. sonnei INV by ELISA (20). During the challenge phase of the study, blood was collected on days 0, 14, and 28 after challenge with 53G (days 60, 74, and 88 for vaccinees after immunization). Significance or seroconversion was defined as a ≥4-fold increase in antibody titers on day 14 and/or 28 after vaccination compared to preimmunization titers (day 0) and on day 14 (day 74 for vaccinees after immunization) and/or day 28 (day 88) after challenge compared to day 0 (day 60). During both phases of the study, fecal samples collected on days 0, 3, 5, and 7 after vaccination and after challenge were analyzed for LPS- and INV-specific IgA antibody titers by ELISA (22). Fecal IgA to LPS and INV was expressed as antibody titers per 100 μg of total IgA (T-IgA) (23). A ≥4-fold increase in fecal IgA titers per 100 μg of T-IgA on days 3, 5, and/or 7 compared to day 0 after vaccination and after challenge was considered a significant response. Antibody titers were determined by a 4-parameter analysis using Soft Max-Pro software (Molecular Devices, Sunnyvale, CA, USA).

### Measurement of ASC and ALS.

During the vaccination phase, blood was collected on days 0, 7, and 9 for measurement of S. sonnei antigens specific for IgA, IgG, and IgM antibody-secreting cells (ASCs) and antibodies in lymphocyte supernatants (ALS). After challenge, blood collection for ASCs and ALS assays followed the same schedule. Peripheral blood mononuclear cells (PBMCs) were isolated from blood samples collected in a BD Vacutainer CPT mononuclear cell preparation tube, and fresh PBMCs were used for ASCs using the enzyme-linked immunosorbent spot (ELISPOT) assay. The ELISPOT assay was used to enumerate IgA, IgG, and IgM ASCs to S. sonnei LPS and S. sonnei INV antigens, and results are expressed as spot-forming units per 10^6^ PBMCs (20). An ASC response of ≥5 was considered significant. For the ALS assay the PBMC concentration was adjusted to 10^7^ PBMCs/ml in complete RPMI medium. One-milliliter aliquots of the PBMCs were cultured in 24-well tissue culture plates at 37°C for 72 h, and the supernatants were harvested and kept frozen and then assayed for IgG and IgA antibodies to S. sonnei antigens by ELISA (23, 24). A ≥4-fold increase in IgA, IgG, and IgM antibody titers in ALS on days 7 and/or 9 compared to day 0 after vaccination and after challenge was considered a significant ALS response.

### Statistical analysis.

Clinical data were analyzed using IBM SPSS Statistics, version 22. Descriptive statistics include geometric means (GMN) for ASC data and geometric mean titers (GMT) for antibody titers with standard errors of the means (SE). Association between postimmunization and postchallenge, clinical signs and symptoms, and excretion and immune responses were assessed by chi-square test (χ^2^) and Fisher's exact test. The Kruskal-Wallis test with Bonferroni adjustment was used to determine the overall significant differences (*P* value of 0.05) of ordinal data among three groups (WRSS1 shedder, WRSS1 nonshedder, and control). If a significant difference was detected, a Mann-Whitney test was performed between any 2 groups; a *P* value of <0.0167 was considered significant. Spearman rank correlation was performed with baseline antibody titers (on day 0 before immunization) and the number of days of shedding of WRSS1 as predictor variables, and peak IgA and IgG ASCs and fold rise in serum IgA and IgG antibody titers were used as outcome variables. Logistic regression was performed with the same predictors along with >40 ASC spots and a 4-fold rise in serum IgA and IgG antibody titers.

## RESULTS

### Volunteers.

A total of 126 Thai adults were screened for this trial (data not shown). Among all screened volunteers, 24% were excluded due to baseline S. sonnei LPS-specific IgG antibody titers that were >800. Except for mean age, there was no statistically significant difference in baseline demographic data among vaccinees and control groups. This included gender, mean height, mean weight, and baseline IgG antibody titers against S. sonnei LPS (data not shown). All enrolled volunteers were monitored until the completion of the study. Volunteers in the first phase of the study received a single oral dose of 1.6 × 10^4^ CFU of WRSS1, and in the second phase the challenge dose of 53G was 1,670 CFU.

### Safety profile of WRSS1 in Thai adult volunteers.

No serious adverse event was reported during the immunization period (Table 1). The vaccine was well tolerated by the 13 volunteers, and none required early antibiotic treatment. Four of 13 vaccinees did not shed the vaccine strain and also did not experience any AE. Nine vaccinees shed the WRSS1 vaccine strain, and 5 of these 9 participants experienced mild AE (characterized by the presence of symptoms but a retained ability to perform daily activities) (Table 1). Two vaccinees had mild diarrhea for a single day that occurred 7 or 9 days after immunization, and another two had mild fever (≤38°C) for a single day that occurred 7 or 8 days after immunization. Since these symptoms occurred 7 days after the single-dose immunization, it is unclear whether these symptoms were related to the effect of the vaccine. One vaccinee had nausea, headache, abdominal pain, and myalgia. All AE were mild and transient in nature. Furthermore, 4 of 6 placebo controls also experienced mild AE (diarrhea, abdominal pain, headache, and rectal tenesmus). Except for the symptom of mild fever, other AEs were similar in vaccinees and placebo controls (Table 1). Thus, although some vaccinees developed objective criteria of illness (fever and diarrhea), these illnesses were mild, were transient, and did not occur in a manner that could clearly be attributed to vaccine administration. No vaccinee had dysentery or fever (≥38.9°C [102°F]) or clinically significant laboratory abnormalities. Therefore, WRSS1 was well tolerated and safe in adult Thai volunteers at 10^4^ CFU.

**TABLE 1:** Clinical symptoms of Thai adults receiving the WRSS1 vaccine or placebo

Group and volunteer serial no.	No. of days of shedding	Presence of disease symptoms^*a*^							No. of individuals with AE
		Clinical		GI		Systemic			
		Diarrhea	Fever	Abdominal pain/cramps	Nausea	Tenesmus	Myalgia	Headache	
Vaccinees									5
1	6	P							
2	8	P							
3	1								
4	9								
5	7		P^*b*^						
6	1								
7^*c*^	6			P	P		P	P	
8^*c*^	11								
9^*c*^	2		P^*b*^						
10	0								
11	0								
12	0								
13	0								
Placebo controls									4
1				P					
2		P							
3				P					
4		P		P		P	P	P	
5									
6									

a P, presence of the symptom. GI, gastrointestinal.

b Seven to 9 days after vaccination, transient.

c Not challenged.

### Fecal shedding of WRSS1.

Fecal shedding of WRSS1 was low by culture, with only 3 of 13 vaccinees (23%) culture positive for 1 to 4 days. Another 6 vaccinees were positive for fecal shedding by PCR, bringing the total number of WRSS1 shedders (WS) to 9 vaccinees (shedding range, 1 to 11 days; median, 6 days; mean, 6 days) (Table 2). As noted above, the remaining 4 vaccinees were in the WRSS1 nonshedder (WNS) group and did not shed the vaccine strain by culture or by PCR.

**TABLE 2:** S. sonnei LPS-specific immune responses in 13 volunteers after WRSS1 vaccination and subsequent S. sonnei 53G challenge

Volunteer serial no.	Postimmunization							Postchallenge^*e*^							Clinical symptom(s) related to challenge
	WRSS1 shedding^*a*^ (days)	ASC count/10^6^ PBMCs^*b*^			Fold increase in^*c*^:			53G shedding^*a*^ (days)	ASC count/10^6^ PBMCs^*b*^			Fold increase in serum Ab titers^*c*^		Fold increase in fecal IgA titers/100 μg of T-IgA^*c*^	
					Serum Ab titers		Fecal IgA titers/100 μg of T-IgA								
		IgA	IgG	IgM	IgA	IgG			IgA	IgG	IgM	IgA	IgG		
1	8	138	30	80	75			5	490	160	130	11			
2	1					4	9	3	530	600		4		8	
3	9	1,290	290	140		4	29	6	6,870	3,980	1,710	64	22	9	Dysentery
4	7	79	23	7			4		144	35	14				Myalgia, headache, malaise^*d*^
5	1	7	5												
6	6		20		6		5								Dysentery
7	0							1	46	14				4	
8	0							2	930	1,000	100	36	20		
9	0							0							
10	0						5	4	4,730	7,480	1,070	169	135		Diarrhea, fever
11	2	12	10												
12	6		8		4	8									
13	11	420	470		4	7									

a Number of days of shedding based on culture and/or PCR of fecal sample.

b Only ASC counts of ≥5 ASCs per 10^6^ PBMCs are shown.

c A ≥4-fold increase in antibody titers was considered significant. Ab, antibody.

d Volunteer serial number 4 had AE but did not meet the criteria of clinical endpoint (diarrhea, dysentery, fever, or shigellosis).

e Volunteers 11, 12, and 13 were not challenged with strain 53G.

### Immune responses after WRSS1 immunization.

**FIG 2:**
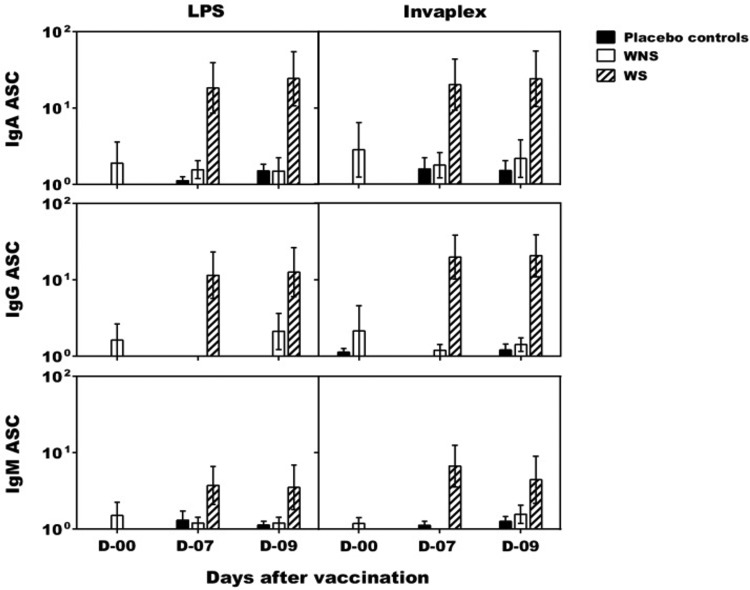
S. sonnei antigen-specific antibody-secreting cell (ASC) counts after oral live S. sonnei vaccine candidate WRSS1 immunization. IgA, IgG, and IgM ASC counts (GMN ± SE) in 13 vaccinees and 6 placebo controls were determined on study days 0, 7, and 9 by ELISPOT assay. Vaccinees were subdivided into WRSS1 shedders (WS) (hatched bar; *n* = 9) and WRSS1 nonshedders (WNS) (white bar; *n* = 4). ASC counts in placebo controls (*n* = 6) are shown as a black bar. Statistical differences between WS and placebo groups were assessed using the Mann-Whitney test: *P* = 0.002 for both LPS- and INV-specific IgA ASC and *P* = 0.009 for both LPS- and INV-specific IgG ASC. In addition, a statistical difference between WS and WNS groups was assessed using the Mann-Whitney test: *P* = 0.006 for both LPS- and INV-specific IgA ASC.

Overall, of 9 WS, 6 (67%) had an IgA ASC, 8 (89%) had an IgG ASC, and 3 (33%) had an IgM ASC response against S. sonnei LPS (Table 2). Similarly, of 9 WS, 7 (78%) had an IgA ASC response, 7 (78%) had an IgG ASC response, and 4 (44%) had an IgM ASC response against S. sonnei INV (data not shown). The WS also displayed LPS-specific IgA and IgG ALS responses (data not shown). There were no ASC responses in the WNS group, indicating a strong relationship between colonization/shedding and demonstration of a mucosal immune response (Fig. 2). As expected, no mucosal immune responses were detected in the placebo controls during the immunization phase of the study. Significant differences were seen between WS and the placebo controls for both LPS- and INV-specific IgA ASC (*P* = 0.002) and IgG ASC (*P* = 0.009). A significant difference was also seen between WS and WNS responses to LPS- and INV-specific IgA ASCs (*P* = 0.006).

**FIG 3:**
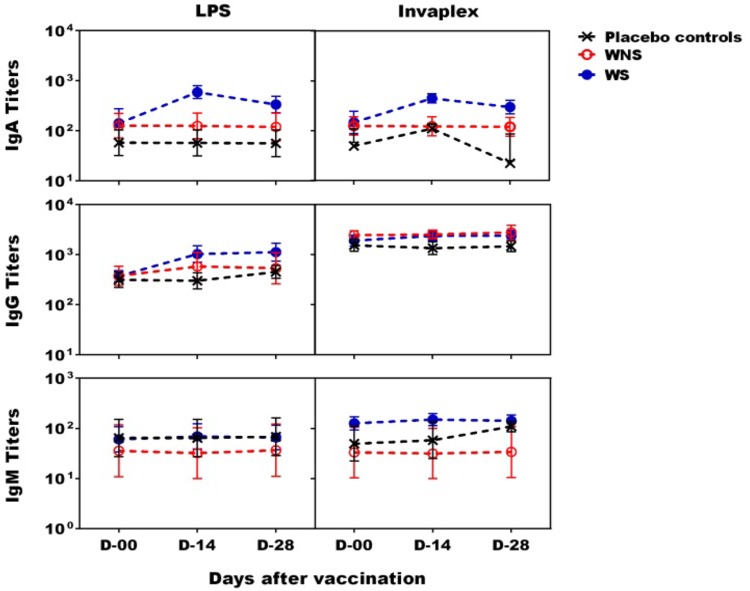
S. sonnei antigen-specific serum antibody titers after oral live S. sonnei vaccine candidate WRSS1 immunization. Serum IgA, IgG, and IgM antibody titers (GMT ± SE) were measured by ELISA for 13 vaccinees and 6 placebo controls on study days 0, 14, and 28. Vaccinees were subdivided into WRSS1 shedder (WS) (blue dotted line; *n* = 9) and WRSS1 nonshedder (WNS) (red dotted line; *n* = 4) groups. Responses of placebo controls (*n* = 6) are shown as black dotted lines. A statistical differences between WS and the placebo groups was assessed using the Mann-Whitney test: *P* = 0.001 for both LPS- and INV-specific IgA titers.

A ≥4-fold increase in LPS- and INV-specific IgA and/or IgG serum antibody titers from the prevaccination day were observed in 6 of the 9 (67%) WS vaccinees (Table 2 and Fig. 3). A significant difference was seen between WS and the placebo controls for both LPS- and INV-specific IgA titers (*P* = 0.001). Three of the 6 WS vaccinees with significant serum antibody titers also demonstrated ≥50 IgA and/or IgG LPS-specific ASC counts. A total of 7 of the 13 vaccinees (including all 4 in the WNS group) failed to demonstrate significant serum antibody titers to either S. sonnei antigen.

A ≥4-fold increase in LPS- and/or INV-specific fecal IgA response was noted in 5 of 9 WS as well as in 2 WNS (Table 2 and data not shown). The geometric mean titers (GMT) of peak fecal IgA per 100 μg of total IgA against both S. sonnei LPS and INV antigens were 9 and 14, respectively, in the WS group and were 8 and 11, respectively, in the WNS group. Surprisingly, 2 of 6 placebo controls also had LPS- or INV-specific fecal IgA responses (data not shown), and one of these two demonstrated mild AE. In the placebo controls the GMT of peak fecal IgA against both LPS and INV antigens was similar to that of the vaccinees (4 and 10, respectively).

### Challenge of WRSS1-immunized volunteers and naive controls with S. sonnei 53G.

Two months after vaccination, 10 randomly selected vaccinees from the first phase of the study along with 10 newly recruited naive controls received ∼1.7 × 10^3^ CFU of virulent S. sonnei 53G that was freshly harvested from TSA plates. Six of the 10 WRSS1-immunized and 53G-challenged vaccinees were WS. In a previous study, the challenge dose that was administered in this study resulted in a dysentery attack rate of 75% (15). In the current study, only 2 of 10 controls suffered from dysentery (attack rate of 20%), including one who had shigellosis (Table 3). After challenge, 4 of 10 vaccinees (40%) (Table 2) and 6 of 10 controls (60%) experienced mild AE (Table 3).

**TABLE 3:** S. sonnei LPS-specific immune responses in naive controls after subsequent S. sonnei 53G challenge

Volunteer serial no.	53G shedding^*a*^ (no. of days)	ASC count/10^6^ PBMCs^*b*^			Fold increase in serum Ab titers^*c*^			Fold increase in fecal IgA titers/100 μg of T-IgA^*c*^	Clinical symptoms related to challenge
		IgA	IgG	IgM	IgA	IgG	IgM		
1	5	2,930	2,810	1,310	15	5			Fever
2	2	6,450	6,760	3,180	13	7			
3	3	1,880	4,770	369	13				Myalgia, fatigue, malaise^*d*^
4	5	2,340	5,140	93	11	7			
5	5	4,390	4,630	1,070	42	23			Shigellosis
6	6	920	620	170	44	43	5		
7	7	1,030	1,150	350	226	50	20		Vomit, diarrhea
8									Dysentery
9								4	Fever^*e*^
10								

a Number of days of shedding based on culture and/or PCR of fecal sample.

b Only ASC counts of ≥5 ASCs per 10^6^ PBMCs are shown.

c A ≥4-fold increase in antibody titers was considered significant. Ab, antibody.

d Control 3 had AE but did not meet the criteria for clinical endpoint (diarrhea, dysentery, fever, or shigellosis).

e Control 9 had IgG and IgM ASC responses to S. sonnei Invaplex (data not shown).

Clinical illness was defined as dysentery, diarrhea, and/or fever. The severity of clinical illness was graded as mild (not affecting normal life), moderate (causing discomfort but not too severely), and severe. After challenge, 3 vaccinees met the clinical criteria for endpoints of disease, with 2 vaccinees having mild dysentery not lasting for more than a day and one vaccinee having mild diarrhea with fever (38.2°C). The 4th vaccinee with AE had only myalgia, headache, and malaise and therefore did not meet the criteria for clinical endpoint. Among naive controls, 5 met clinical endpoints of disease, with one having severe shigellosis, one having mild dysentery, one having diarrhea and vomiting, and 2 volunteers having fever (one had 38.8°C, one had 38.2°C). The 6th naive control with AE had only myalgia, fatigue, and malaise (did not meet the criteria for the clinical endpoint). Therefore, based on clinical record summaries, a vaccine efficacy (for Shigella-related illness) of 40% was calculated with the formula vaccine efficacy = (disease rate in naive controls − disease rate in vaccines)/disease rate in naive controls × 100. Although there was no efficacy against dysentery and diarrhea, there was 66% efficacy against fever (based on fever in 3 naive controls and in 1 vaccinee).

### Fecal shedding after 53G challenge.

There was greater shedding after challenge with 53G than after immunization with WRSS1 (Tables 2 and 3). Stools from 3 of the 6 WS vaccinees were culture positive for 53G (*n* = 3; shedding range, 2 to 4 days; mean, 3 days) and RT-PCR positive for 53G (*n* = 3; shedding range, 3 to 6 days; mean, 5 days). Stools from 3 of 4 WNS vaccinees also were culture positive for 53G (*n* = 3; shedding range, 1 to 3 days; mean, 2 days) and RT-PCR positive for 53G (*n* = 3; shedding range, 1 to 4 days; mean, 2 days). Stools from 7 of 10 naive controls after challenge with 53G were culture positive for the strain (*n* = 7; shedding range, 1 to 6 days; mean and median, 3 days) and RT-PCR positive for 53G (*n* = 7; shedding range, 1 to 7 days; median, 5 days; mean, 4 days). The durations of 53G fecal shedding were not significantly different between vaccinees and naive controls (Tables 2 and 3). However, among the three naive controls that did not shed 53G, 2 had dysentery, fever, and abdominal pain (Table 3). Among the 4 vaccinees that did not shed 53G, one had myalgia, headache, and malaise and one had dysentery (Table 2).

### Immune responses after 53G challenge.

**FIG 4:**
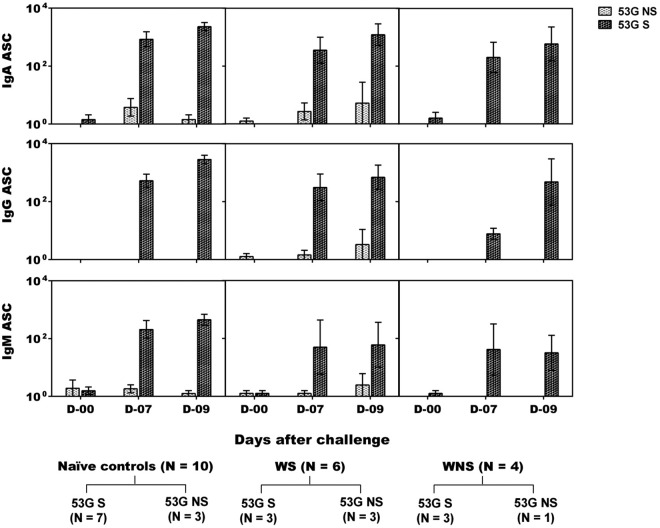
S. sonnei LPS-specific ASC counts in oral live S. sonnei vaccine candidate WRSS1-immunized vaccinees and control volunteers after wild-type S. sonnei 53G challenge. IgA, IgG, and IgM ASC counts (GMN ± SE) were determined on study days 0, 7, and 9 for naive controls (*n* = 10) and on study days 0 (60), 7 (67), and 9 (69) for WRSS1-immunized vaccinees (*n* = 10) after 53G challenge. Ten vaccinees were subdivided into WRSS1 shedders (WS) (*n* = 6) and WRSS1 nonshedders (WNS) (*n* = 4). Within each group, 53G shedders (53G S) and 53G nonshedders (53G NS) are represented as dark spotted bars and light spotted bars, respectively.

**FIG 5:**
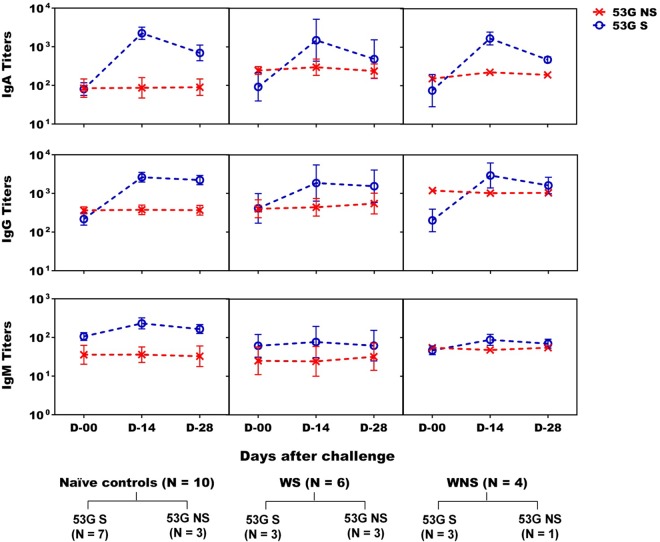
S. sonnei LPS-specific serum antibody titers in oral live S. sonnei vaccine candidate WRSS1-immunized vaccinees and control volunteers after wild-type S. sonnei 53G challenge. Serum IgA, IgG, and IgM antibody titers (GMT ± SE) were measured on study days 0, 14, and 28 for naive controls (*n* = 10) and on study days 0 (60), 14 (74), and 28 (88) for WRSS1-immunized vaccinees (*n* = 10) after 53G challenge. Ten vaccinees were subdivided into WRSS1 shedder (WS) (*n* = 6) and WRSS1 nonshedder (WNS) (*n* = 4) groups. Within each group, 53G shedders (53G S) and 53G nonshedders (53G NS) are represented as blue dotted lines and red dotted lines, respectively.

Seven of 10 vaccinees challenged with 53G developed significant ASC responses to S. sonnei antigens, with 6 volunteers having >100 LPS-specific IgA ASCs. Six of the 7 vaccinees who developed ASCs also shed the challenge strain (Table 2 and Fig. 4). Coincidentally, of these 7 vaccinees, 5 demonstrated serum IgA, 3 demonstrated IgG responses to S. sonnei antigens, and 2 demonstrated IgM responses to S. sonnei INV (Table 2 and Fig. 5). In this respect, the connection between shedding and development of immune responses bears similarity to that seen during the immunization phase of the study. In the naive control group, 7 of 10 participants shed 53G and elicited high IgA, IgG, and IgM ASC responses as well as significant serum IgA and IgG responses to S. sonnei antigens (Table 3 and Fig. 4 and 5). Robust ALS responses also were detected (not shown). Two of the three nonshedding naive controls did not demonstrate mucosal or serum antibody responses. The third nonshedding naive control demonstrated ASC and fecal IgA responses to INV and also developed fever (Table 3).

Fecal IgA responses to S. sonnei antigens were seen in only 3 of 10 vaccinees after 53G challenge. Among the 10 challenged controls, only one showed fecal IgA responses to LPS and two had responses against INV. In the controls who shed the 53G strain, the GMT of LPS- and INV-specific peak fecal IgA titers per 100 μg of total IgA was 1 and 2, respectively, compared to 16 and 28, respectively, in controls that did not shed the 53G strain. A similar trend was seen in WRSS1-immunized volunteers. Challenged vaccinees who shed WRSS1 and 53G had lower fecal IgA titers against LPS and INV (3 and 8, respectively) than those that shed WRSS1 but did not shed 53G (58 and 29, respectively). In volunteers who did not shed WRSS1 but shed 53G, the GMT of peak fecal IgA titers against LPS and INV were 7 and 22, respectively. The single individual who did not shed WRSS1 as well as 53G had fecal IgA antibody titers against both LPS and INV of 17 and 16, respectively.

### Correlates of immune response.

Postimmunization data were analyzed for possible correlates of protection and immune responses. Spearman rank correlation showed that the duration of fecal shedding of WRSS1 correlated with increased serum IgA titers to LPS (*P* = 0.01), fold increases in serum IgA (*P* = 0.00) and IgG (*P* = 0.03) levels, and an increase in the peak number of anti-LPS IgA ASCs (*P* < 0.00) and IgG ASCs (*P* = 0.00) compared to the baseline. The duration of fecal shedding (at least 6 days) also correlated with >40 IgA ASC spots (odds ratio [OR], 27; *P* = 0.009) and a 4-fold increase in IgA antibody titers (OR = 55; *P* = 0.009) by logistic regression. Among the 13 vaccinees, 4 had >50 IgA, IgG, and/or IgM ASC spots (Table 2). Among these 4 vaccinees, one was not selected for challenge, one had no AE after challenge, and one had myalgia, malaise, and headache after challenge. The fourth vaccinee (volunteer no. 3 in Table 2), a WRSS1 and 53G shedder, had high levels of humoral and mucosal immune responses postimmunization and postchallenge but nonetheless suffered mild dysentery, nausea, insomnia, dizziness (on the 3rd day after challenge), and malaise on the 4th day after challenge.

## DISCUSSION

This is the first efficacy study with WRSS1 and was performed in a population of adults in an area where the organism is endemic and where S. sonnei currently predominates over other serotypes of Shigella. Data from U.S. and Israeli trials indicated that WRSS1 was well tolerated at doses of 10^3^ and 10^4^ CFU, where mild and transient diarrhea and fever were seen in some volunteers. At higher doses, the rate of diarrheal and febrile reactions increased, suggesting that 10^4^ CFU would be a suitable dose for future trials in these populations. Therefore, a single dose of 10^4^ CFU was used to immunize Thai adults, which proved to be safe and well tolerated (18, 19, 25–27). However, since higher doses and repeated doses were not evaluated in this study, it is uncertain whether 10^4^ CFU was the optimal dose for eliciting maximal immunogenicity in this adult population from a region where the organism is endemic. During prescreening, 24% of the volunteers demonstrated >800 LPS-specific serum IgG antibody titers, with a GMT of 382, which is slightly higher than what was found in Israelis (25). One reason for preexisting titers may be the prevalence of Plesiomonas shigelloides in Thailand. P. shigelloides has an O-antigen structure identical to that of S. sonnei (26, 27). Volunteers with S. sonnei LPS-specific serum antibody titers of >800 were excluded from the clinical study. However, although the existence of LPS-specific serum antibody responses during prescreening is used as a marker for prior exposure to Shigella, 30% of the control volunteers in this study who were challenged with 53G did not elicit a serum antibody response (seroconversion), even though two of these three volunteers suffered from clinical endpoints of disease and one demonstrated a mucosal response in the form of INV-specific ASCs and fecal IgA. Thus, lack of serum antibody titers may not always be reflective of a lack of prior exposure.

Although the dose of 10^4^ CFU of WRSS1 was safe and well tolerated in healthy Thai adults, 5 of 13 (38%) vaccinees as well as 4 of 6 (67%) placebo controls reported mild AE, which included mild and transient diarrhea in both groups and mild and transient fever in the vaccinee group. The fever in vaccinees was seen 7 to 9 days after vaccination and therefore may not be related to vaccine administration. One volunteer had to be excluded from the study on day 0 due to diarrhea. Acute diarrhea is a common problem among outpatients in Thailand (28). As a result, it is sometimes difficult to assess vaccine-induced symptoms in small groups of adult volunteers from areas in which the pathogen is endemic and among whom diarrheal episodes are frequent.

Clinical endpoints of disease were defined as shigellosis, diarrhea, dysentery, or fever. In this study, 3 of 10 (30%) vaccinees challenged with 53G met the criteria for the clinical endpoints, although the AEs were mild except for one case of moderate malaise. Among the controls, 5 of 10 participants (50%) met the criteria for clinical endpoints: 4 participants had mild AE, including 1 case of dysentery, 1 case of diarrhea with vomiting, and 2 cases of fever with or without diarrhea, and 1 participant had severe AE (shigellosis). While efficacy against single endpoints of disease was not evident in this study, based on the number of controls and vaccinees who demonstrated clinical endpoints after challenge, a trend in protection could be seen after WRSS1 immunization. In a previous study, administration of 1,680 CFU of 53G resulted in a 75% attack rate of dysentery in control volunteers, with 100% of volunteers having abdominal symptoms and 1 of 12 volunteers having shigellosis (20). In the current study, a low attack rate of dysentery (20%) in the naive controls prevented a statistically significant assessment of vaccine efficacy.

Data from previous studies with WRSS1 indicated that strong immune responses follow robust colonization of the vaccine strain as monitored by excretion. In this study, vaccinees that did not shed WRSS1 did not elicit immune responses. Similarly, after challenge, naive controls and vaccinees that did not shed 53G had lower immune responses than those that shed the challenge strain. However, lack of shedding appeared to correlate with higher fecal IgA titers, suggesting that local immunity played a part in limiting colonization. In the U.S. trials, there was clear evidence of intestinal vaccine replication, as demonstrated by recovery of 10^4^ to 10^7^ CFU of WRSS1 per g of stool in 82% of the vaccinees compared to only 23% of Thai adults who were culture positive for the vaccine. In the U.S. study, culture-positive fecal shedding occurred over a period of 5 to 7 days up to initiation of antibiotic treatment. Shedding was dose dependent, with 50% of the U.S. and Israeli volunteers shedding the vaccine strain at a lower dose (3 × 10^3^ CFU) and 100% of the volunteers shedding at the highest tested dose (3 × 10^6^ CFU). In a community-based evaluation of WRSS1 in Israel, more than 70% of the vaccinees excreted the vaccine for a period ranging between 1 and 23 days (19). It is unclear what factors contribute to the low levels of vaccine shedding in Thai adults. Prior exposure to the organism in an area where it is endemic may lead to serotype-specific immunity in adults and may lead to a low priming effect of the vaccine candidate (29).

The level of resistance to colonization of WRSS1 in Thai adults is similar to what was observed with another oral live attenuated Shigella vaccine candidate, SC602 (S. flexneri 2a), in Bangladesh (30). Such a resistance to infection has been observed with other oral vaccines, such as polio, cholera, and rotavirus vaccines, leading to the administration of higher doses and repeated vaccine doses (15). Perhaps a dose determination study is needed to optimize the dose for colonization in a population where the disease is endemic, and volunteers may have different levels of innate immunity to the particular pathogen. Such an optimal dose determination study, as well as defining the role of repeated doses, could improve the outcome of WRSS1 vaccination in Thai adults. A better understanding of the gut microbiome in different populations before and after vaccination/challenge may be required to determine if the gut microbiome plays a role in determining the uptake of an oral vaccine.

Currently there are no absolute correlates of protection for assessment of Shigella vaccines. Natural clinical Shigella infections confer ∼75% protection against a homologous serotype. Protection is thought to be due to local adaptive humoral responses mediated by secretory IgA (sIgA), the main immunoglobulin found at the mucosal surface (25, 31–36). Protection has also been achieved by passively transferred type-specific oral immunoglobulin (37), suggesting that serum (38) and mucosal antibodies play a central role in preventing shigellosis. WRSS1 induced strong mucosal and systemic immune responses in U.S. volunteers reminiscent of the responses seen after immunization and challenge with SC602. Vaccinees immunized with a single dose of 10^4^ CFU of SC602 and challenged 2 months later with a virulent S. flexneri 2a strain demonstrated 100% protection against moderate to severe diarrhea, dysentery, and fever. In the SC602 study, a 3- to 4-fold increase in serum IgA/IgG/IgM titers coupled with >40 IgA ASCs against S. flexneri 2a LPS appeared to correlate with protection, although the study was performed in a limited number of volunteers. Since Shigella is a mucosal pathogen, a mucosal immune response is expected to be part of protection, perhaps in combination with an antibody response.

An important aspect of the immune response that is missing from the earlier studies is the determination of the functionality of serum antibodies as well as the T and B cell phenotypes that are driving the mucosal response (39, 40). Bactericidal assays and opsonophagocytosis measurements may provide a correlation between functional serum antibodies and protection and should be carried out in future trials. Furthermore, other factors need to be investigated, such as the role of gamma interferon, interleukin-17 (IL-17), and IL-22 production, as well as production of B memory cells with α4β7^+^ integrins after Shigella vaccination (31, 32, 39).

In summary, a single oral dose of 10^4^ CFU of S. sonnei vaccine candidate WRSS1 was safe in Thai adults and generated significant humoral and mucosal immune responses. Challenge of immunized volunteers with virulent S. sonnei strain 53G indicated a trend toward protection that could be improved by higher doses and repeated doses of the vaccine candidate. A clear correlation was observed between vaccine shedding as well as shedding of the challenge strain and subsequent elicitation of immune responses.
